# Salmonella enterica Serovar Typhi Lipopolysaccharide O-Antigen Modification Impact on Serum Resistance and Antibody Recognition

**DOI:** 10.1128/IAI.01021-16

**Published:** 2017-03-23

**Authors:** Erica Kintz, Christian Heiss, Ian Black, Nicholas Donohue, Naj Brown, Mark R. Davies, Parastoo Azadi, Stephen Baker, Paul M. Kaye, Marjan van der Woude

**Affiliations:** a Centre for Immunology and Infection, Hull York Medical School and Department of Biology, University of York, York, United Kingdom; b Complex Carbohydrate Research Center, The University of Georgia, Athens, Georgia, USA; c The Hospital for Tropical Diseases, Wellcome Trust Major Overseas Programme, Oxford University Clinical Research Unit, Ho Chi Minh City, Vietnam; d Centre for Tropical Medicine, Oxford University, Oxford, United Kingdom; University of California, Davis

**Keywords:** O-antigen, Salmonella enterica, lipopolysaccharide, phase variation, serum resistance

## Abstract

Salmonella enterica serovar Typhi is a human-restricted Gram-negative bacterial pathogen responsible for causing an estimated 27 million cases of typhoid fever annually, leading to 217,000 deaths, and current vaccines do not offer full protection. The O-antigen side chain of the lipopolysaccharide is an immunodominant antigen, can define host-pathogen interactions, and is under consideration as a vaccine target for some Gram-negative species. The composition of the O-antigen can be modified by the activity of glycosyltransferase (*gtr*) operons acquired by horizontal gene transfer. Here we investigate the role of two *gtr* operons that we identified in the *S*. Typhi genome. Strains were engineered to express specific *gtr* operons. Full chemical analysis of the O-antigens of these strains identified *gtr*-dependent glucosylation and acetylation. The glucosylated form of the O-antigen mediated enhanced survival in human serum and decreased complement binding. A single nucleotide deviation from an epigenetic phase variation signature sequence rendered the expression of this glucosylating *gtr* operon uniform in the population. In contrast, the expression of the acetylating *gtrC* gene is controlled by epigenetic phase variation. Acetylation did not affect serum survival, but phase variation can be an immune evasion mechanism, and thus, this modification may contribute to persistence in a host. In murine immunization studies, both O-antigen modifications were generally immunodominant. Our results emphasize that natural O-antigen modifications should be taken into consideration when assessing responses to vaccines, especially O-antigen-based vaccines, and that the Salmonella *gtr* repertoire may confound the protective efficacy of broad-ranging Salmonella lipopolysaccharide conjugate vaccines.

## INTRODUCTION

Salmonella enterica subsp. enterica serovar Typhi is responsible for an estimated 27 million new cases of typhoid fever and 217,000 deaths annually (1). Infection with *S*. Typhi occurs via the fecal-oral route; after ingestion, bacteria cross the intestinal epithelium, enter the bloodstream, and spread systemically (2). In some cases, *S*. Typhi is capable of colonizing the gallbladder, leading to chronic asymptomatic shedding and contributing to the infection cycle. Two vaccines are currently licensed for use against *S*. Typhi: the parenterally administered Vi capsular polysaccharide subunit vaccine and the orally administered live-attenuated Ty21a vaccine. Neither vaccine offers complete protection (3), and there is ongoing research into new vaccine formulations against *S*. Typhi and other Salmonella infections (3, 4). Conjugate vaccines combining carrier proteins with the Vi polysaccharide antigen are under development. However, Vi expression can be up- or downregulated, and Vi-negative isolates have been isolated from typhoid patients (5, 6). Lipopolysaccharide (LPS) is a Gram-negative bacterial virulence factor, is a component of the outer membrane, and, in the absence of Vi, is the predominant *S*. Typhi surface carbohydrate. Notably, the efficacy of the *S*. Typhi Ty21 vaccine is associated in part with the expression of LPS (7).

LPS is composed of a lipid A tail, which anchors the LPS into the membrane; a core oligosaccharide; and an O-antigen side chain. The surface-exposed O-antigen side chain protects the bacterial cell from the actions of the innate immune system (8). The O-antigen is immunogenic and may be a functional target for novel vaccines (9, 10). The S. enterica subspecies is comprised of over 2,600 serovars, which are based on differences in the antigenic properties of the O- and H (flagellar)-antigens and form the basis of the Kauffman-White serotyping system (11). During natural infection, antibodies are raised against LPS, and the detection of *S*. Typhi O-antigen antibodies forms the basis of the diagnostic Widal test for typhoid (12). Furthermore, there is significant cross-reactivity between serovars sharing certain O-antigen epitopes, which may be exploited for the development of pan-Salmonella vaccines (13). Therefore, gaining insight into the occurrence and significance of variation in *S*. Typhi O-antigen composition may enhance the understanding of *S*. Typhi pathogenesis and support the development of diagnostic and intervention tools and therapies.

Modifications of LPS, including those in the O-antigen, play a role in many chronic bacterial infections (14). O-antigen structures can be modified through several processes, and recently, we identified and characterized numerous Salmonella *gtr* (glycosyltransferase) operons (15). Using the amino acid sequence identity of the GtrC O-antigen-modifying proteins, we were able to group the *gtr* operons into 10 different “families” and proposed that each family performs a different O-antigen modification (15). We additionally noted that a single Salmonella isolate may harbor multiple *gtr* operons, and several families of these *gtr* operons can undergo phase variation (15, 16), thus generating further potential complexity of the O-antigen presented by a population. If, as a result, clonal bacterial populations have a nonuniform O-antigen composition, this could serve as a means of immune evasion (17–19).

The significance of *gtr*-mediated O-antigen modification for Salmonella biology is not fully understood. In S. enterica serovar Typhimurium, specific modifications have been implicated in gut colonization (20) and in phage resistance (21). To better understand the extent and impact of O-antigen variation, we aimed to characterize the activity and expression of the *gtr* repertoire in *S*. Typhi. Given that *S*. Typhi causes systemic infection and that evasion of the innate immune response can contribute to persistence, we measured the effect of *gtr* modification on serum sensitivity. Furthermore, we assessed the antibody response to each O-antigen modification in serum in a murine immunization model.

## RESULTS

The genomes of *S*. Typhi strains Ty2, CT18, and P-stx-12 (isolated from a chronic carrier in India) (22) each contain two different *gtr* operons (15). These operons share sequence identity between the *S*. Typhi strains. One operon is a family 3 *gtr* type with high identity (99% amino acid) to the *S*. Typhimurium family 3 operon (STM0557 to STM0559) that mediates the α1→4 glucosylation of the O-antigen galactose sugar (20). The second *gtr* operon could be grouped with the family 2 GtrCs and shared 77% amino acid identity with a similar operon in invasive *S*. Typhimurium isolate D23580. This GtrC operon has been hypothesized to acetylate the rhamnose residue of the O-antigen (21); the *S*. Typhi O-antigen has a rhamnose residue, but no acetylation has been described.

**FIG 1:**
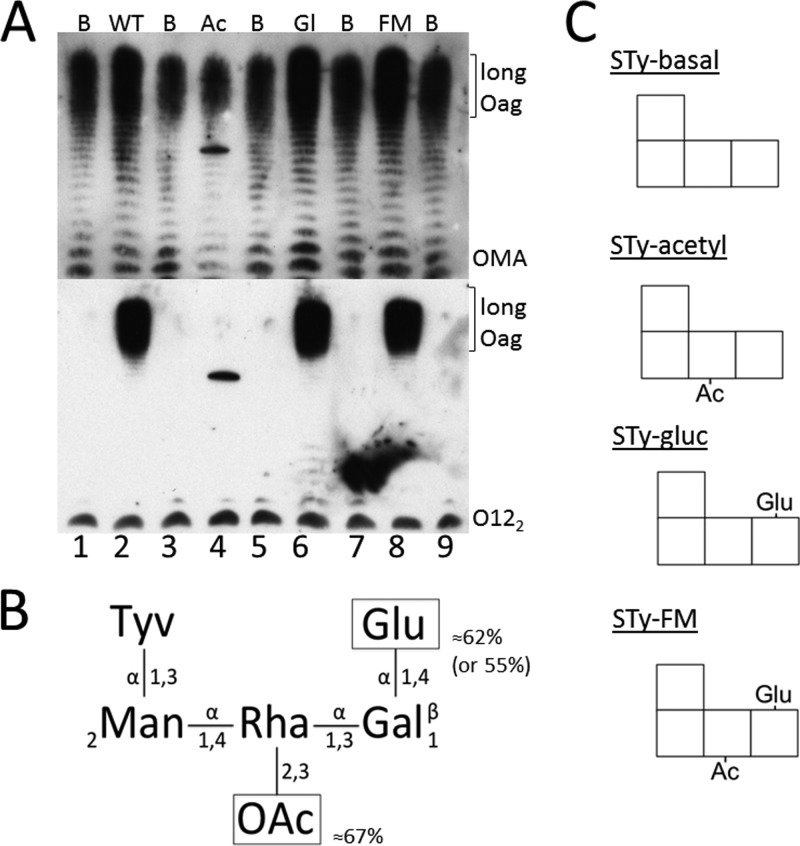
Effect of *gtr* modifications on *S*. Typhi O-antigen. (A) Expression of the family 3 *gtr* operon leads to recognition by O12_2_ serum. LPS was prepared as described in Materials and Methods and run on a Tricine–SDS-PAGE gel. Blots were probed with commercial OMA serum (top) and O12_2_ serum (bottom). B, STy-Basal; WT, *S*. Typhi parent strain BRD948 (“wild type”); Ac, STy-Acetyl; G, STy-Gluc; FM, STy-FM; Oag, O-antigen. (B) Summary of results from chemical analysis of *S*. Typhi BRD948 O-antigen. OAc, acetate. (C) Schematic of the O-antigens of engineered strains used in these studies. See Fig. S1 in the supplemental material for additional silver stain analysis of the *S*. Typhi O-antigen.

To assess the role of the *S*. Typhi *gtr* operons, we generated a set of four otherwise isogenic *S*. Typhi strains with a defined *gtr* expression pattern: STy-Basal (both *gtr* operons deleted), STy-Acetyl (expressing only family 2), STy-Gluc (expressing only family 3), and STy-FM (both *gtr* operons expressed). LPSs from these isogenic strains were extracted and compared by Western blotting (Fig. 1; see also Fig. S1 in the supplemental material). The O-antigens of all strains reacted with commercial Salmonella serum, confirming that all strains expressed O-antigen (Fig. 1A) and that the production of the long antigen structure was not affected. Factor O12_2_ serum targets the α1-4 glucosylation of the galactose (23), and the O-antigens of the parent *S*. Typhi strain, STy-Gluc, and STy-FM reacted with this serum; strains lacking the expression of the family 3 *gtr* operon did not react with this serum (Fig. 1A). Silver staining showed that strains expressing the family 3 *gtr* operon had a distinct O-antigen laddering pattern compared to that of isolates that lacked the family 3 *gtr* operon (Fig. S1). These data indicate that the family 3 *gtr* operon of *S*. Typhi performs the same O-antigen modification as the family 3 *gtr* operon of *S*. Typhimurium, namely, α1-4 glucosylation of the galactose. However, no visible shift in the O-antigen pattern for STy-Acetyl compared to STy-Basal was observed, providing further evidence that family 2 GtrC does not act as a glucosyltransferase.

### Chemical analysis of the O-antigens from *S*. Typhi strains.

To define the compositions and linkages of the *gtr*-dependent modifications, various chemical analyses were performed on LPSs isolated from the *S*. Typhi parent strain and isogenic variants. Full details are available in Fig. S2 and S3 and Tables S2 to S4 in the supplemental material. A methylation analysis of the LPSs of these strains showed only minor differences in the proportions of the linkages present in the polysaccharide (Table 1). The *S*. Typhi parent strain and both STy-Gluc and STy-FM showed glucosylation on *O*-4 of galactose at 62.3%, 82.4%, and 82.5%, respectively, deduced from the galactose linkages. Nuclear magnetic resonance (NMR) analysis revealed that the extent of glucosylation was comparable to that derived from the methylation analysis (Table S2 and Fig. S2) (55.9% for the parent, 72.8% for STy-Gluc, and 71.2% for STy-FM).

**TABLE 1:** Methylation analysis of the three LPS samples^*a*^

Modification	Glycosyl linkage	mol%		
		BRD948	STy-Gluc	STy-FM
a	*t*-Tyv	0.2	2.3	3.0
b	4-Rha	13.2	15.4	17.2
c	*t*-Glc	17.6	15.2	19.4
d	2-Man or 3-Man	1.8	3.0	3.2
e	3-Gal	6.8	3.6	3.3
f	4-Glc	24.7	20.4	19.7
g	2,3-Man	15.6	16.2	15.9
h	3,4-Gal	11.3	16.8	15.6
i	4,6-Glc	1.5	1.6	0.8

a The DG (degree of glucosylation) values [*h*/(*h* + *e*) × 100] were 62.3% for BRD948, 82.4% for STy-Gluc, and 82.5% for STy-FM.

In contrast to the methylation analysis, in which *O*-acetyl groups are removed during acid hydrolysis, NMR analysis of untreated LPS (no lipid A removal) permits the identification of acetylation. We recorded an O-acetylation signal from untreated LPSs of both the parent *S*. Typhi strain and STy-FM (both harbor family 2 *gtr* operons) (Fig. S2), but this signal was absent in the spectra from STy-Gluc. A further evaluation identified a 4-linked 3-*O*-acetyl rhamnose and a 4-linked 2-*O*-acetyl-rhamnose (Fig. S3 and Table S4). The two states likely reflect a single modification event, with subsequent migration of the acetyl group (24). The parent *S*. Typhi strain exhibited the same pattern of peaks in all NMR spectra as that exhibited by the STy-FM strain, with only slight disparities in the intensities of the acetylated positions (50% and 67%, respectively) and with *O*-2 and *O*-3 of rhamnose in approximately equal abundances. Taken together, these data confirm that the *S*. Typhi family 3 *gtr* operon mediates the α1→4 glucosylation of the galactose and show that the family 2 *gtr* operon acts as a rhamnose acetyltransferase (Fig. 1B and C).

### The expression patterns of the two *S*. Typhi *gtr* operons differ.

**FIG 2:**
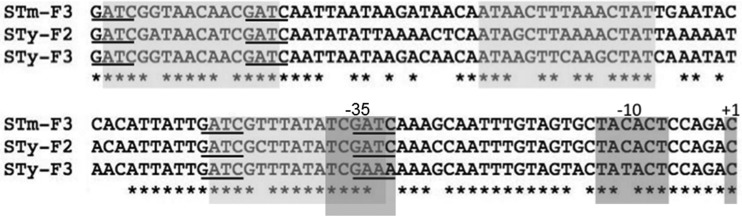
Alignment of *S*. Typhi *gtr* regulatory regions to the phase-varying regulatory region of the *S*. Typhimurium family 3 *gtr* operon. The GATC sites are underlined, and the OxyR binding sites are outlined in light gray boxes. The −35/−10 sigma sites and the +1 transcriptional start site are indicated. Alignment was performed by using TCOFFEE.

The expression of multiple *gtr* operons in S. enterica is controlled by phase variation (16). This regulation is associated with a signature sequence in the regulatory region of *gtrA* comprised of two binding sites for the transcriptional regulatory protein OxyR and four GATC sites. The GATC sites are the target sequence for Dam, a “maintenance” adenine DNA methyltransferase. The DNA methylation-dependent interaction of OxyR at the *gtr* binding sites leads to epigenetic phase variation of *gtr* expression (15, 16). In *S*. Typhi, this signature sequence is present in the family 2 *gtr* regulatory region, and therefore, we predicted that this operon undergoes phase variation. In contrast, the family 3 operon has the OxyR binding sequences but only three GATC sequences, with GAAA at the fourth, promoter-proximal, GATC sequence (Fig. 2).

The expression of both *gtr* operons was assessed by using single-copy transcriptional *lacZ* fusions. In the *S*. Typhimurium and *S*. Typhi backgrounds, family 2 expression was controlled by phase variation. The switch frequency of the on phase to the off phase was similar to that for the reverse switch (Table 2). Therefore, in the absence of selective pressure, a clonal population should consist of similar numbers of cells with and without the family 2-mediated modification. In contrast, the strain with the *lacZ* reporter for the family 3 *gtr* operon gave rise to only Lac^+^ colonies, indicating that the *S*. Typhi family 3 *gtr* operon is expressed but not controlled by phase variation. Mutation of GAAA back to GATC restored phase variation to this family 3 *gtr* operon (data not shown). These data indicate that a clonal *S*. Typhi population is likely to have a uniform O-antigen glycosylation pattern but is heterogeneous at the single-cell level with respect to O-antigen acetylation.

**TABLE 2:** Expression of S. Typhi *gtr* operons^*b*^

Strain	Lac phenotype	Miller units (SD)^*a*^	On-to-off switch frequency	Off-to-on switch frequency
LT2^F2reg-lacZ^	Lac^+^/Lac^−^	1,116 (42)	3.0 × 10^−3^	3.3 × 10^−3^
LT2^F3reg-lacZ^	Lac^+^	763 (28)	NA	NA

a Standard deviations are shown in parentheses. Miller units were calculated for 100% switched-on cells for phase-varying isolates.

b NA, not applicable.

### Effect of *gtr* modifications on serum sensitivity.

*S*. Typhi spreads systemically during typhoid fever; therefore, the infecting organisms must have reliable mechanisms for survival in the presence of components of the innate immune system. The O-antigen can contribute to serum survival. We next assessed whether *gtr* expression and its effects on O-antigen composition affect serum resistance in *S*. Typhi.

**FIG 3:**
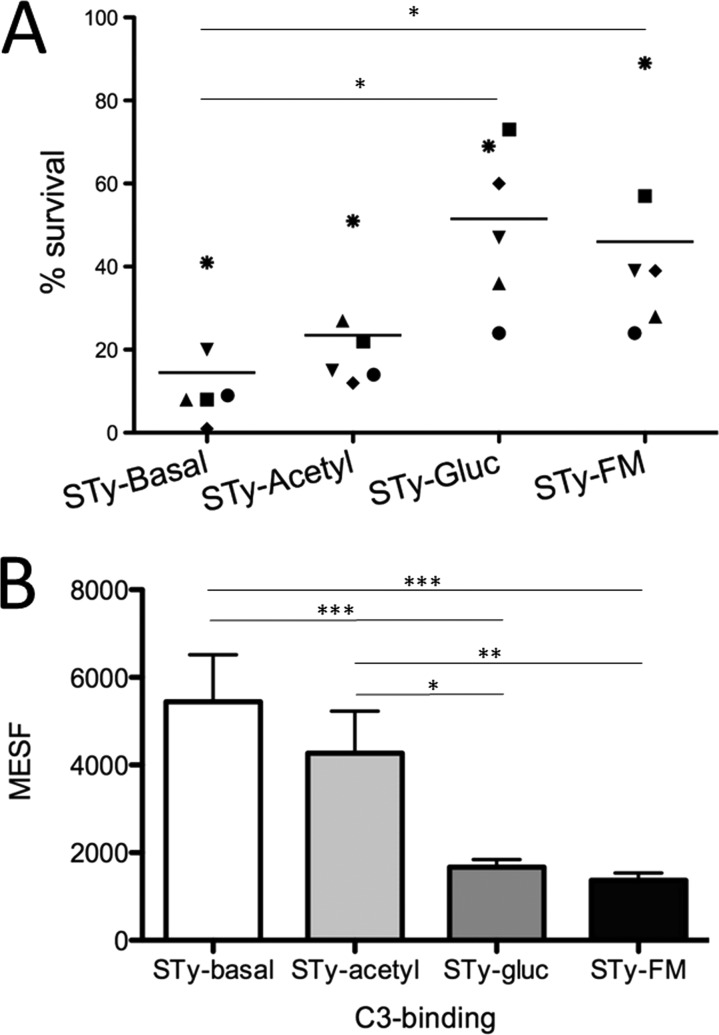
Serum sensitivity of *S*. Typhi strains with different *gtr* modifications. (A) Results from the serum survival assay for strains grown to stationary phase and incubated in 50% serum. Different symbols represent the data obtained from individual experiments. One-way analysis of variance and Tukey postanalysis were performed by using GraphPad Prism (version 5.0d). *, *P* < 0.05. (B) Binding of C3 protein to *S*. Typhi strains with different O-antigen compositions. Strains were incubated in human serum, followed by anti-C3 FITC-conjugated antibody, and surface-bound fluorescence was measured by flow cytometry. Data from four experiments are combined. MESF, molecules of equivalent soluble fluorochrome. One-way analysis of variance and Tukey postanalysis were performed by using GraphPad Prism (version 5.0d). *, *P* < 0.05; **, *P* < 0.01; ***, *P* < 0.001. Error bars indicate means ± standard errors of the means.

The serum sensitivities of the various isogenic *S*. Typhi strains were measured by using commercially available human serum (Fig. 3A). The two strains containing family 3-mediated glucosylation had greater resistance to serum killing than did the STy-Basal and STy-Acetyl strains, both of which lack glucosylation. Acetylation of the O-antigen did not afford any significant survival benefit compared to STy-Basal under any of the conditions tested. Taken together, these results imply that complement-mediated killing of *S*. Typhi is altered by the *gtr*-dependent glucosylation of the O-antigen.

To corroborate differences in serum sensitivity between glucosylated and nonglucosylated strains, we next assessed the binding of the C3 complement protein to the strains. C3 initiates alternate pathway activation, leading to the formation of the membrane attack complex (MAC). After incubation in naive human serum, cells were exposed to fluorescein isothiocyanate (FITC)-conjugated anti-C3 antibody, and the level of surface-bound C3 was measured by flow cytometry. Nonglucosylated strains (STy-Basal and STy-Acetyl) exhibited significantly more surface-bound fluorescence than did glucosylated strains (STy-Gluc and STy-FM) (Fig. 3B). Acetyl modification did not significantly alter C3 binding (comparisons of STy-Basal to STy-Acetyl and of STy-Gluc to STy-FM).

### Recognition of *S*. Typhi O-antigen from a murine immunization model.

**FIG 4:**
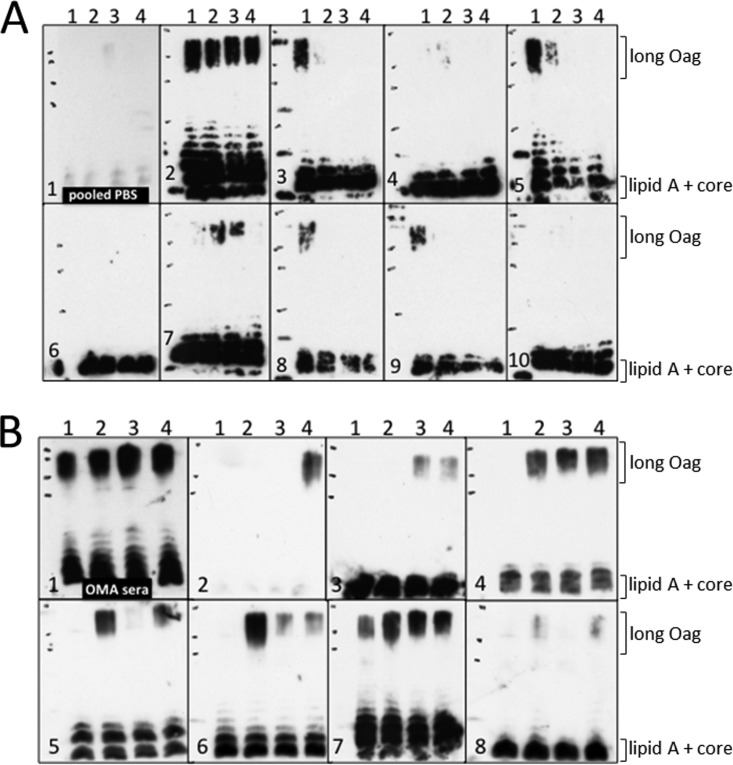
Recognition of *S*. Typhi O-antigen by serum in a murine immunization model depends on the O-antigen composition of the immunizing strain. In an LPS Western blot, LPSs from different strains were probed with serum from mice immunized with either Sty-Basal (A) or STy-FM (B). Panel A1 shows the reactivity of pooled serum from the PBS-immunized control group, and each following panel (panels 2 to 10) represents serum from an individual mouse. Panel B1 shows the reactivity of commercial OMA serum, which recognizes several Salmonella serovars; each following panel represents serum from an individual mouse. Lanes 1 to 4 in each panel show LPS from the following strains, respectively: STy-Basal, STy-Acetyl, STy-Gluc, and STy-FM.

We assessed whether O-antigen modification affected the specificity of antibodies generated in a murine immunization model. Mice were immunized with either STy-Basal (no O-antigen modification) or STy-FM (acetylated and glycosylated O-antigen), and serum was collected. LPSs from the four strains with defined *gtr* expression were used in a Western blot assay and probed with serum from individual mice (Fig. 4). This approach allowed the identification of the O-antigen moieties recognized by the antibody and discrimination from the antibody directed to the shared lipid A and core.

Irrespective of the immunizing strain, there were variations between the responses of individual mice (Fig. 4A and B). Immunization with nonmodified O-antigen uniformly resulted in the recognition of lipid A and core (Fig. 4A). Some animals (3/9) (e.g., Fig. 4A10) failed to show any additional recognition of the O-antigen structures. When an O-antigen response was evident, the composition of O-antigen from the immunizing strain was most frequently recognized (5/9) and, in some cases, was the only O-antigen that was recognized (3/9) (e.g., Fig. 4A3) Notably, only one serum sample recognized all four forms of *S*. Typhi O-antigen (Fig. 4A2).

In contrast, in mice immunized with the STy-FM strain expressing a fully modified O-antigen, the unmodified form of O-antigen was recognized in only 1/7 mice (Fig. 4B7), suggesting a general immunodominance of the *gtr*-dependent modifications (Fig. 4B).

All three modified O-antigen forms were recognized in only 3/7 mice (e.g., Fig 4B4). The remaining mice produced antibodies specific for either the glucosylated or the acetylated form despite having been immunized with a strain expressing both modifications. These data predict that *gtr*-mediated modifications are mostly immunodominant over the unmodified form (Sty-Basal) but that neither acylation nor glucosylation is preferentially recognized after immunization with a strain expressing O-antigen with both modifications.

## DISCUSSION

The occurrence of strain- and serovar-dependent O-antigen modification in the salmonellae has long been recognized (25), but only recently has the potential for *gtr*-mediated modification been described for this genus (15). Here we investigated two *gtr* operons in the human host-restricted pathogen *S*. Typhi. Our data show that the family 3 *gtr* operon catalyzes α1→4 glucosylation of galactose, as was previously described for a family 3 *gtr* operon of *S*. Typhimurium (20). Additionally, we demonstrate that the family 2 *gtr* operon is required for the acetylation of rhamnose, as was suggested previously (21). Thus, family 2 GtrC is not a glycosyltransferase but an acyltransferase, and the *gtr* acronym thus reflects only proximity to (remnant) *gtrAB* genes (15). Galactose glucosylation in the *S*. Typhi O-antigen was previously reported (25), but, to our knowledge, this is the first report of rhamnose acetylation in *S*. Typhi. Acetylation of the family 3-dependent glucose modification may also occur, but the genes encoding this process remain to be identified (25, 26).

Acetylation of the rhamnose moiety will be heterogeneous in a bacterial population due to phase variation of the expression of the family 2 *gtr* operon. This is consistent with a role in, and a mechanism of, immune evasion (19). In contrast, the STy family 3 *gtr* regulatory region deviates from the known phase variation signature sequence in one GATC sequence, which caused an abrogation of phase variation. Thus, glucosylation should be uniformly expressed among cells in a population, likely contributing to the high degree of glucosylation observed in *S*. Typhi O-antigen compared to those of other Salmonella serovars (26, 27). The sequence variation associated with this lack of phase variation is present in both the *S*. Typhi CT18 and Ty2 genomes.

O-antigen glucosylation has implications for virulence in nontyphoidal serovars. In *S*. Typhimurium, family 3-dependent glucosylation is associated with increased persistence in the mouse intestine (20), and glucosylation of the S. enterica serovar Enteritidis O-antigen is associated with an increase in virulence in a chicken-to-egg transmission model (27). The role that we identified for family 3-dependent glucosylation in serum resistance adds to the evidence that O-antigen glucosylation can affect Salmonella-host interactions (14, 28). This finding does not exclude further benefits of this modification for *S*. Typhi.

Antibody recognition of a pathogen is an important feature of the clearance of infection. In *S*. Typhi, the Vi capsular polysaccharide contributes to immune evasion (29), and antigen O9 antibodies can affect antibody-mediated serum resistance. This role of O9 antibodies may be relevant for antibody-mediated killing when there is reduced Vi capsule expression during the infection cycle (30, 31). Our data highlight that both *gtr*-mediated O-antigen modifications can influence antibody recognition of the O-antigen. The trend from the murine model was that immunization with a strain with modified O-antigen generated antibodies that predominantly recognized the modified version of Salmonella O-antigen and did not recognize the basal, unmodified O-antigen. These modifications thus could impact antibody-dependent killing mechanisms during *S*. Typhi infection.

In other Salmonella serovars, dominant epitopes induced by O-antigen modification have been shown to be relevant for eliciting a protective immune response. For example, OafA-dependent acetyl modification of abequose is required for protective antibodies against *S*. Typhimurium (32, 33). A protective S. enterica serovar Paratyphi A glycoconjugate LPS vaccine required acetylation, which is likely dependent on the described rhamnose acetylation modification (34, 35). Based on the data presented here linking rhamnose acetylation to the family 2 *gtr* operon, this can now be attributed to the family 2 *gtr* operon harbored by the *S*. Paratyphi A genome (15). Rhamnose acetylation was also implicated in the strain-specific dominant epitope of the invasive *S*. Typhimurium D23580 isolate (33), which can be attributed to the phage-borne family 2 *gtr* operon that is expressed in this specific strain (21). However, detailed analyses in the context of the host, disease, and serovars are needed to further clarify the impact of O-antigen acetylation and glucosylation (36, 37).

Our results expand the body of evidence demonstrating that O-antigen composition in Salmonella impacts host-pathogen interactions during infection. Strains within a specific serovar may have different repertoires of O-antigen-modifying genes, and expression may fluctuate by phase variation. Consequently, antibody generated by primary infection or immunization may not wholly recognize subsequent infections by the same serovar. Indeed, disease-associated *S*. Typhi strains are not clonal, and the genetic repertoire of O-antigen-modifying genes may vary (15, 38, 39). Furthermore, the Ty21a oral vaccine elicits a strong O-antigen antibody response (13), and thus, any factors that modify this response may impact serovar and strain cross-reactivity. In conclusion, we suggest that O-antigen modification repertoires may need to be considered in vaccine design to enhance efficacy against a broad range of *S*. Typhi isolates and phenotypes.

## MATERIALS AND METHODS

### Bacterial strains and culture conditions.

Strains were grown in LB unless mentioned otherwise. For *S*. Typhi BRD948, medium was supplemented with an Aro mix (final concentrations of 40 μM l-phenylalanine, 40 μM l-tryptophan, 1 μM *para*-aminobenzoic acid, 1 μM 2,3-dihydroxybenzoic acid, and 40 μM tyrosine). The following antibiotics were used: tetracycline at 15 μg/ml, ampicillin at 100 μg/ml, chloramphenicol at 34 μg/ml for vectors and 8 μg/ml for chromosomal inserts, and kanamycin at 30 μg/ml. *S*. Typhi BRD948 and its derivatives were confirmed to be Vi positive (Vi^+^) by serum agglutination (Vi antiserum; Statens Serum Institute). For analysis of LacZ expression, strains were grown on minimal M9 medium (Sigma, Gilingham, UK) with 0.2% glucose (*S*. Typhimurium) or LB (*S*. Typhi) with 40 μg/ml X-gal (5-bromo-4-chloro-3-indolyl-β-d-galactoside; Melford). Strains are listed in Table S1 in the supplemental material.

### Molecular biology, strain construction, and mutagenesis.

Standard molecular biology techniques were used (40). Details on vectors and primers used in these studies are provided in Table S1 in the supplemental material. Strains containing a *lacZ* reporter fusion on the chromosome at the *attB* site were generated by using the CRIM (conditional-replication, integration, and modular) system (41), using vector pMV243 (42). Allelic replacement was used to introduce mutations into the chromosome (43). Antibiotic resistance cassettes were obtained from Tn*10* for Tc^r^ and pKD4 for Km^r^ (43). Unmarked strains were generated by the removal of the pKD13-derived Km^r^ cassette by Flp recombinase expressed from pCP20 (44). Vectors and primer details are provided in Table S1.

### Analysis of gene expression.

β-Galactosidase assays on strains with *lacZ* reporter fusions were performed as described previously by Miller (45). Cultures derived from two independent colonies were grown in M9 minimal medium with glucose. Samples were collected at least in triplicate between optical density at 600 nm (OD_600_) values of 0.3 to 1.5 for measurement of β-galactosidase activity, given in Miller units. The switch frequency of phase variation was calculated for two independent colonies each for a Lac^+^ or Lac^−^ phenotype, as described previously (46). The switch frequency is expressed as the number of cells that have a changed expression state (*M*) over the total number of cells (*n*), divided by the number of generations (*g*).

### LPS extraction and visualization.

Crude LPS extracts were prepared as described previously (15) and separated on a Tricine–SDS-PAGE gel, and O-antigen was visualized by using a silver stain (15) or Western blotting. These crude extracts can incidentally contain contaminating macromolecules that are apparent on the gels or Western blots. For Western blot analyses, samples were transferred onto a polyvinylidene difluoride (PVDF) membrane and blocked with 5% milk in phosphate-buffered saline-Tween (PBS-T). A O12-OMA polyvalent antiserum mixture for salmonella O-antigen from groups A, B, D, E, and L (Statens Serum Institute) or O12_2_ serum was used to probe blots as indicated, using secondary goat anti-rabbit IgG-horseradish peroxidase (HRP) (catalog number A0545; Sigma). When mouse serum was used, goat anti-mouse IgG-HRP (catalog number A1068) served as a secondary antibody. The Luminato Western HRP substrate (Millipore) was used for detection.

### Serum sensitivity assay.

Human serum was purchased from Sigma (catalog number H4522). This serum failed to agglutinate the *S*. Typhi strains. *S*. Typhi strains were grown overnight and then diluted to an OD_600_ of 0.5 in PBS. Five microliters of bacteria was added to 45 μl of serum diluted in PBS, and the mixture was incubated for 1 h at 37°C. Dilutions were performed to determine CFU. Survival was determined by comparing counts to those obtained from a control that was incubated in 45 μl PBS. Experiments were performed in duplicate and repeated at least three times.

### C3 binding to the bacterial surface.

Experiments were performed by using stationary-phase bacteria. Methods were adapted from those described previously by MacLennan et al. (47), using quantities similar to those used in the serum sensitivity experiments. Briefly, bacteria were incubated with full-strength human serum followed by incubation with polyclonal rabbit anti-human C3c FITC antibody (catalog number F-0201; Dako). A Beckman Coulter CyAn ADP analyzer was used to collect data. A Bangs FITC-5 MESF (molecules of equivalent soluble fluorochrome) kit (catalog number 555) allowed the standardization of fluorescence across experiments.

### Generation of murine immune serum.

Female *Slc11a1*^D169^ (Nramp1^s^) C57BL/6 CD45.1 mice were obtained from Charles River UK, housed under specific-pathogen-free conditions, and used at 6 to 10 weeks of age. All experiments were approved by the University of York Animal Welfare and Ethical Review Body and performed under a UK Home Office license. For immunizations, bacteria from cultures grown overnight were diluted to an OD_600_ of 1.0 in PBS. Two hundred microliters was given intraperitoneally (i.p.), resulting in an inoculum ranging from 8.12 × 10^7^ to 2.56 × 10^8^ CFU. Twenty-eight days after immunization, mice were exsanguinated by cardiac puncture under terminal anesthesia. Serum was obtained by allowing the collected blood to coagulate overnight at 4°C and then centrifuging the blood at 2,000 rpm for 2 min. The serum layer was then removed and stored at −20°C until use. Nine mice were immunized with the STy-Basal strain, and seven were immunized with the STy-FM strain.

## Supplementary Material

Supplemental material
