# Supplementary material for: Salmonella enterica Serovar Typhi Lipopolysaccharide O-Antigen Modification Impact on Serum Resistance and Antibody Recognition
Source: Infect Immun. 2017 Mar 23;85(4):e01021-16. doi: 10.1128/IAI.01021-16 (PMC5364305; doi:10.1128/IAI.01021-16)
Supplement: Supplemental material [file supp_85_4_e01021-16__index.html]

Supplemental material 

# Salmonella enterica Serovar Typhi Lipopolysaccharide O-Antigen Modification Impact on Serum Resistance and Antibody Recognition

## Supplemental material

- Supplemental file 1 -

  Supplemental methods. Table S1. Strains, vectors, and primers used. Table S2. Chemical shift assignments of the de-O-acetylated *S.* Typhi OPS samples. Table S3. Relative intensities of the main anomeric signals in the three de-O-acetylated OPS samples. Table S4. Chemical shift assignment of the acetylated residues found in *S.* Typhi LPS 623 and 707. Fig. S1. Effect of *gtr* modification on mobility of *S.* Typhi O antigen on an LPS Tricine SDS-PAGE gel. Fig. S2. Anomeric region of the 1-D proton spectra of the three de-O-acetylated OPS samples. Fig. S3. NMR analysis of *S.* Typhi LPS.

  PDF, 648K
