# Supplementary material for: Salmonella enterica Serovar Typhi Lipopolysaccharide O-Antigen Modification Impact on Serum Resistance and Antibody Recognition
Source: Infect Immun. 2017 Mar 23;85(4):e01021-16. doi: 10.1128/IAI.01021-16 (PMC5364305; doi:10.1128/IAI.01021-16)
Supplement: Supplemental material [file IAI.01021-16_zii999092015s1.pdf]

The chemical modification of the *S*. Typhi O-antigen mediated by the two *gtr* families.

The following pages include methods and additional results for the NMR analysis, Tables S1-S4, Figures S1-S3.

Material and Methods

Preparation of samples for NMR

Isolation was performed as described by Brett et al. (1), but acetic acid hydrolysis was omitted in order to avoid loss of O-acetylation. Briefly, strains were grown 0.5L of LB media supplemented with Aro/Tyr mix and ampicillin overnight and pelleted. Pellets were resuspended in 250 ml 90% ethanol and left for one hour at room temperature to kill the bacteria. Bacteria were pelleted again, washed in 50 ml water and resuspended in 10 mls water. The samples were frozen and vacuum freeze-dried for three days. The resulting material (about 0.5 g total) was processed further for chemical analysis. Full details on LPS isolation and the chemical analysis can be found in the supplemental Materials and Methods.

LPS isolation for chemical analysis

The sample was suspended in 10 mM tricine buffer (pH 8.0) containing 1 mM $CaCl_2$ and 5 mg/mL proteinase K and incubated for 2 d at room temperature. The solution was then centrifuged at 100,000 g overnight. The supernatant was discarded, and the pellet was dried and extracted using phenol/water. The dried sample was transferred into glass tubes and re-suspended in 5 mL water. The suspension was heated to 65 °C and vigorously stirred. Phenol was then added to the suspension, and the mixture was stirred for 1 h. Then the sample was cooled on ice and centrifuged at 3,000 g. The aqueous phase was removed, and the phenol phase was washed once with water. The combined aqueous phases were dialyzed using 1kDa MWCO dialysis membranes. The phenol phase was dialyzed separately to remove the phenol. The dialyzed aqueous phase was centrifuged at 100,000 g overnight, and the supernatant was discarded. The pellet was lyophilized and the dry material was used for the composition analysis.

Methylation analysis

The sample was permethylated, depolymerized, reduced, and acetylated, and the resultant partially methylated alditol acetates (PMAAs) were analyzed by gas chromatography-mass spectrometry (GC-MS) using a modification of the method described by York et al.(2)

About 1 mg of each sample was suspended in 200 µl DMSO. The samples were permethylated using 400 µL a suspension of NaOH in DMSO (3). After stirring for 15 min, 50 µL CH_3I was added, and the mixture was left for 45 min. The addition of NaOH and CH_3I was repeated to ensure complete methylation of the polymer. After an additional 45 min, 2 mL water was added, and excess CH_3I was removed by sparging with nitrogen gas. The mixture was extracted with CH_2Cl_2, and after washing the organic phase 3 times with water and drying it down with nitrogen gas, the permethylated material was hydrolyzed using 2 M trifluoroacetic acid (2 h in sealed tube at 121 °C), reduced with $NaBD_4$, and acetylated using acetic anhydride/trifluoroacetic acid. The resulting PMAAs were analyzed on a Agilent 7890NA GC interfaced to a 5975BC MSD (mass selective detector, electron impact ionization mode). Separation was achieved on a 30 m Supelco 2380 bonded phase fused silica capillary column.

For mild acid hydrolysis, the samples were heated in 1% acetic acid at 100 °C for 1 h. The lipid fraction was removed by centrifugation at 10,000 g, and the supernatant was freeze-dried. For de-*O*-acetylation, the samples were dissolved in water (10 mg/mL), and the solution was brought to pH 11 by addition of concentrated ammonia water. After allowing the reaction to proceed at room temperature for 18 h, the solution was dialyzed (2 kDa) against water and freeze-dried.

<u>NMR Spectroscopy</u>

For de-O-acetylated polysaccharide analysis, the samples were deuterium exchanged by lyophilization from D_2O (99.9% D, Aldrich) and dissolved in 300 µL D_2O (99.96% D, Cambridge Isotopes). One-dimensional proton and 2-D gCOSY, TOCSY, NOESY, gHSQC, and gHMBC spectra were obtained on a Varian Inova-600 MHz spectrometer at 70 °C using standard Varian pulse sequences. TOCSY and NOESY mixing times were 80 and 300 ms, respectively. Chemical shifts were measured relative to internal acetone (δ_H=2.218 ppm, δ_C=33.0 ppm) (4). To analyze native LPS, the samples were deuterium exchanged by dissolving in D_2O and lyophilization and dissolved in 700 µL D_2O (some material did not dissolve). 1-D proton and 2-D gCOSY, TOCSY, NOESY, HMQC (or gHSQC), and gHMBC (STy-

Gluc and STy-FM only) spectra were obtained on a Varian Inova-600 MHz spectrometer at 70 °C using standard Varian pulse sequences. TOCSY and NOESY mixing times were 80 and 300 ms, respectively. Chemical shifts were measured relative to the residual HDO signal (δ=4.31 ppm at 70 °C), using a Ξ value of 0.25144953 for ^{13}C.

Results of NMR analysis

Differences in glucosylation

We obtained the de-*O*-acetylated *O*-chains of the three LPS samples by mild acid hydrolysis to cleave off Lipid A and ammonium hydroxide saponification to remove *O*-acetyl groups. We performed NMR in order to test whether the mutations entailed any differences in the monosaccharide sequence of the *O*-chain repeating unit. We were particularly interested to know if there was a difference in the DG (degree of glucosylation) on *O*-4 of the galactose residue. The 1-D proton spectra of the de-*O*-acetylated polysaccharides were almost identical in all three samples, but we did notice some differences in the intensity of some of the minor anomeric signals (Figure S2, Peaks G, K, I, and M). In order to identify these peaks, we obtained 2-D COSY, TOCSY, NOESY, HSQC, and HMBC NMR spectra of the de-*O*-acetylated samples in order to assign most of the signals, hoping that this would allow us to measure the DG. We were able to identify the spin systems related to the presence or absence of the α-Glc residue, most notably 3-linked and 3,4-linked α-Gal, but also two versions each of 4-α-Rha and 2,3-α-Man (see Table S2). Two additional Tyv anomeric signals were observed as well. Unexpectedly, we also detected a β-Gal residue that was 3-linked to another 4-Rha and may represent a defect in the polysaccharide chain. Using the connectivities obtained from the NOESY and HMBC spectra, we were able to group these residues into 3 subunits, namely →2)[α-Tyv-(1→3)]-α-Man-(1→4)-α-Rha-(1→3)[α-Glc-(1→4)]-α-Gal-(1→ (Subunit I), →2)[α-Tyv-(1→3)]-α-Man-(1→4)-α-Rha-(1→3)-α-Gal-(1→ (Subunit II), and →2)[α-Tyv-(1→3)]-α-Man-(1→4)-α-Rha-(1→3)-β-Gal-(1→ (Subunit III). Subunits I and II have been reported previously (5, 6), but Subunit III has not. With the completed assignments in hand, it seemed possible to quantify the 3-Gal and 3,4-Gal residues in order to obtain the DG in each sample, but the Gal residues were not resolved in either the 1-D proton or the 2-D HSQC spectra. Nevertheless, the Rha residue of each

subunit was resolved from the others, and they could be used to measure the overall DG. The intensities of the anomeric signals and the calculated DG are listed in Table S3. This analysis showed that BRD948 had a significantly lower DG (55.9%) than both STy-Gluc (72.8%) and STy-FM (71.2%).

Differences in acetylation

To determine whether the mutations had an effect on the polysaccharide *O*-acetylation pattern, the LPS samples were analyzed by NMR as intact LPS, without prior lipid A removal to avoid loss of *O*-acetyl groups through hydrolysis. *O*-acetylation was indeed detected in the NMR spectra of both untreated LPS for the BRD948 and STy-FM samples, as evidenced by signals at 2.16 and 2.14 ppm, but. These signals had a carbon resonance in HSQC at 23.1 ppm, in agreement with the expected chemical shift for an acetyl-CH_3 group. The acetylated positions were recognized in HSQC by their downfield proton resonances (5.17 and 5.02 ppm) and distinguished from anomeric signals by their upfield carbon resonances (75.3 and 75.6 ppm). No acetylation was seen in the spectra of the STy-Gluc sample. Comparison of the TOCSY spectra of STy-Gluc and STy-FM showed that the latter had two additional rhamnose residues, labeled B' and B". This was especially clear from the cross peaks correlated to H-6 of the rhamnose residues (Figure S3A and S3B). H-6 of Residue B" (1.34 ppm) showed a cross peak to the acetylated position at 5.02 ppm. Tracing the connectivities in COSY (not shown), TOCSY, and HSQC (Figure S3C) allowed identification of this signal as H-3 of a 4-linked 3-*O*-acetyl rhamnose (Table S4). The TOCSY spectrum did not have a cross peak directly connecting H-6 of B' (1.32 ppm) and the other acetylated position (at 5.17 ppm). However, H-6 did show correlations with peaks at 3.88, 3.56, and 4.02 ppm, and these in turn correlated with the acetylated position at 5.17 ppm. The proton resonances and carbon chemical shifts of Residue B', obtained from HSQC, identified it as 4-linked 2-*O*-acetyl-rhamnose (Table 4). BRD948 showed the same peak pattern in all NMR spectra as STy-FM, only the intensities of the acetylated positions were slightly different. The intensities of the two acetyl signals in both samples (1.5 for STy-FM and 2.0 for BRD948) suggested degrees of acetylation of about 50 and 67%, respectively. The acetylation of *O*-2 and *O*-3 of rhamnose appeared to be of approximately equal abundance.

Table S1. Strains, vectors, and primers used

Strains

Strain Name	Strain Number	Relevant Genotype	Plasmid	Source
Escherichia coli **isolates**				
NEB5a				NEB
	MV1017	pir+		
	MV382	Tn10 source		
Salmonella **Typhi isolates**				
BRD948	sMV623	BRD948 (Ty2 ΔaroC aroD htrA)		(7)
STyΔFam2	sMV638	ΔFam2		This study
RegTet	sMV656	ΔFam2; Fam 3 promoter::tet		This study
tetKO	sMV657	ΔFam2; Fam3::tet		This study
STy-basal	sMV682	ΔFam2; Fam3::tet	pLAC22	This study
STY-acetyl	sMV708	ΔFam2; Fam3::tet	pMV412	This study
STy-gluc	sMV662	ΔFam2; Fam 3 promoter::tet	pLAC22	This study
STy-FM	sMV707	ΔFam2; Fam 3 promoter::tet	pMV412	This study
BRD948$^{F3reg\text{-}lacZ}$	sMV668	BRD948 *attB*::Fam3 promoter-*lacZ*	*attB*::pMV389	This study
BRD948$^{23reg\text{-}lacZ}$	sMV667	BRD948 *attB*::Fam2 promoter-*lacZ*	*attB*::pMV389	This study
Salmonella **Typhimurium isolates**				
JR501	sMV150			(8)
LT2	sMV77			ATCC
LT2$^{F3reg\text{-}lacZ}$	sMV636	LT2 *attB*::Fam 3 promoter-*lacZ*	*attB*::pMV389	This study
LT2$^{23reg\text{-}lacZ}$	sMV637	LT2 *attB*::Fam2 promoter-*lacZ*	*attB*::pMV388	This study

Vectors

Name	Parent Plasmid	Description; gene cloned with oligonucleotides used	Source
pKD4		FRT flanked kanR template	(9)
pCP20		Helper plasmid FRT, temperature sensitive	(10)
pINT-ts		Helper plasmid λ-integrase, temperature sensitive	(11)
pSIM18		Helper plasmid λ-red, temperature sensitive	(12)

Oligonucleotides

oMV number	Sequence (5' to3')[a]	Description
oMV968	GCACCTGCAGTAACAACAAGTTATC	PstI; Fam3 promoter region
oMV969	GGGTACCCTTCAACATTATGAAAATC	Acc651; Fam3 promoter region
oMV970	CATGCTGCAGTTTTTAACGCTCCCCCTCTC	PstI; Fam2 promoter region
oMV971	CATGGGTACC<br>CTTCAACATTATGAAAATTAGCCG	Acc651; Fam2 promoter region
RKS562	TGTAGTGCTACACTCCAGACCTTTCTGAATCGG<br>CTAATTTTC ATA *CATATGAATATCCTCCTTAG*	Fam2::Kan *gtr*A start
RKS563	CTGGCATGTTCCATATTGGATTTATATGTAATAG<br>TAACAGCC TAT *GTGTAGGCTGGAGCTGCTTCG*	Fam2::Kan *gtr*C end
oMV977	CACTCAGCAATCAGCAGCTCCAATTGATCGGTA<br>ACAACG *CTAAGCACTTGTCTCCTG*	Fam 3::tet *gtr*A start
oMV496	TATTTCCGTAATATTCTCATTTGTCCTCGCCCCTG<br>TTCTAACGTCCCATGT<br>*TTAAGACCCACTTTCACATT*	Fam 3::tet *gtr*C end
oMV972	TAACAACAAGTTATCCCATTACCACGGCGCTCA<br>CTCGCC *TTAAGACCCACTTTCACATT*	Fam 3 promoter::tet
oMV973	AACACCGATCGATGTGTACTTAGCGAATAACTT<br>CAACAT *CTAAGCACTTGTCTCCTG*	Fam 3 promoter::tet
oMV776	TACTATAGATCTATGTTGAAGTTATTCGCTAAGT<br>AC	BglII; Cloning pMV412 (*gtr*A start)
oMV780	ATTAGTGAATTCCTATTTGATTATTTTATTTCCG	EcoRI; Cloning pMV412 (*gtr*C end)

a. Restriction enzymes used are underlined and text in italics is homologous to the antibiotic resistance cassette

Supplemental Table S2. Chemical shift assignments of the de-O-acetylated *S*. typhi OPS samples. Carbon chemical shifts are in italics. Three different subunits were found, differing in substitution by α-Glc and anomeric configuration of 3-Gal.

Sub-uni	No.	Residue	Chemical shift (ppm) 1	2	3	4	5	6
**I**	**A**	2,3-α-Man	5.228	4.03	4.04	3.95	3.95	3.83/3.77
			103.2	*81.2*	*79.7*	*69.0*	*76.0*	*63.3*
	**B**	4-α-Rha	5.159	4.05	3.84	3.56	3.85	1.33
			104.1	*73.2*	*71.8*	*84.9*	*70.8*	*19.8*
	**C**	3,4-α-Gal	5.252	4.08	4.01	4.14	4.08	3.83/3.79
			103.6	*71.8*	*77.9*	*79.7*	*75.1*	*62.9*
	**D**	α-Tyv	4.917	4.05	2.05/1.81	3.61	3.75	1.26
			103.6	*69.9*	*36.2*	*69.4*	*73.2*	*19.3*
	**E**	α-Glc	5.000	3.53	3.76	3.55	3.88	3.87/3.81
			102.7	*74.6*	*75.1*	*71.8*	*74.6*	*63.8*
**II**	**F**	2,3-α-Man	5.258	4.03	4.04	3.95	3.95	3.83/3.77
			102.2	*81.2*	*79.7*	*69.0*	*76.0*	*63.3*
	**G**	4-α-Rha	5.082	4.07	3.95	3.56	3.92	1.32
			104.6	*73.2*	*71.8*	*84.5*	*70.9*	*19.8*
	**H**	3-α-Gal	5.228	3.95	3.93	ND	4.03	3.83/3.79
			103.6	*71.8*	*77.9*	*nd*	*74.1*	*62.9*
	**I**[a]	α-Tyv	4.906	4.05	2.05/1.81	3.61	3.75	1.26
			103.6	*69.9*	*36.2*	*69.4*	*73.2*	*19.3*
**III**	**J**	2,3-α-Man	ND	4.01	3.90	3.84	ND	ND
			ND	*81.2*	*80.7*	*69.0*	*ND*	*ND*
	**K**	4-α-Rha	5.068	4.07	3.95	3.56	3.92	1.32
			104.6	73.2	*71.8*	*84.5*	*70.9*	*19.8*
	**L**	3-β-Gal	4.526	3.69	3.70	3.99	3.73	ND
			105.5	*73.2*	*83.0*	*72.7*	*77.9*	*ND*
	**M**[a]	α-Tyv	4.884	4.04	2.05/1.87	3.63	3.75	1.26
			103.6	*69.9*	*36.2*	*70.4*	*73.2*	*19.3*

[a]These two residues may be interchanged

Supplemental Table S3. Relative intensities of the main anomeric signals in the three de-O-acetylated OPS samples.

No.	Residue	Mole per cent		
		**662**	**623**	**707**
**A** + **B**	3,4-α-Gal +2,3-α-Man	19.5	18.8	16.1
**C** + **D**	2,3-α-Man + 3-α-Gal	17.2	17.7	18.7
**F**	4-α-Rha-(A)	16.4	16.3	17.4
**G**	4-α-Rha-(D)	2.6	6.5	3.9
**L**	4-α-Rha-(M)	3.5	6.3	3.2
**I**	α-Glc[a]	18.9	12.7	18.5
**K**	Tyv	18.2	16.3	19.0
**M**	3-β-Gal	3.7	5.3	3.2
DG[b]		72.8	55.9	71.2

[a]The Glc signal is high because there are two other residues overlapping, one of which may be 3-α-Man.

[b]DG=(F/(F+G+L)*100)

Supplemental Table S4. Chemical shift assignment of the acetylated residues found in *S. typhi* LPS 623 and 707 (carbon chemical shifts in italics)

No.	Residue	Chemical shift (ppm)					
		**1**	**2**	**3**	**4**	**5**	**6**
**B’**	4-(2-OAc)-α-Rha[a]	5.14	5.17	4.02	3.56	3.88	1.32
		101.3	*75.3*	*70.0*	*84.8*	*71.1*	*19.8*
**B”**	4-(3-OAc)-α-Rha[a]	5.10	4.15	5.02	3.77	3.96	1.34
		104.0	*70.7*	*75.6*	*81.0*	*70.9*	*20.3*

[a]O-acetate: 2.16/23.1, 2.14/23.1 ppm

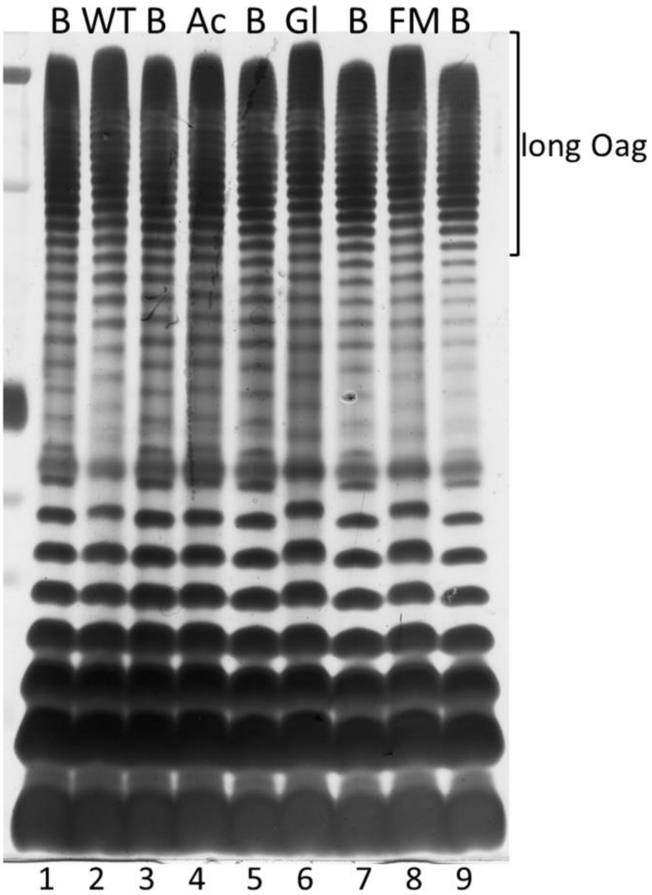

Figure S1. The effect of *gtr* modification on mobility of S. Typhi O-antigen on a LPS Tricine SDS-PAGE gel. LPS was visualized with silver staining. Shifts become evident 4-7 O-antigen bands up from the bottom of the gel. B = Sty-basal; WT = wild-type BRD948; AC = STy-acetyl; Gl = STy-gluc; FM = STy-FM.

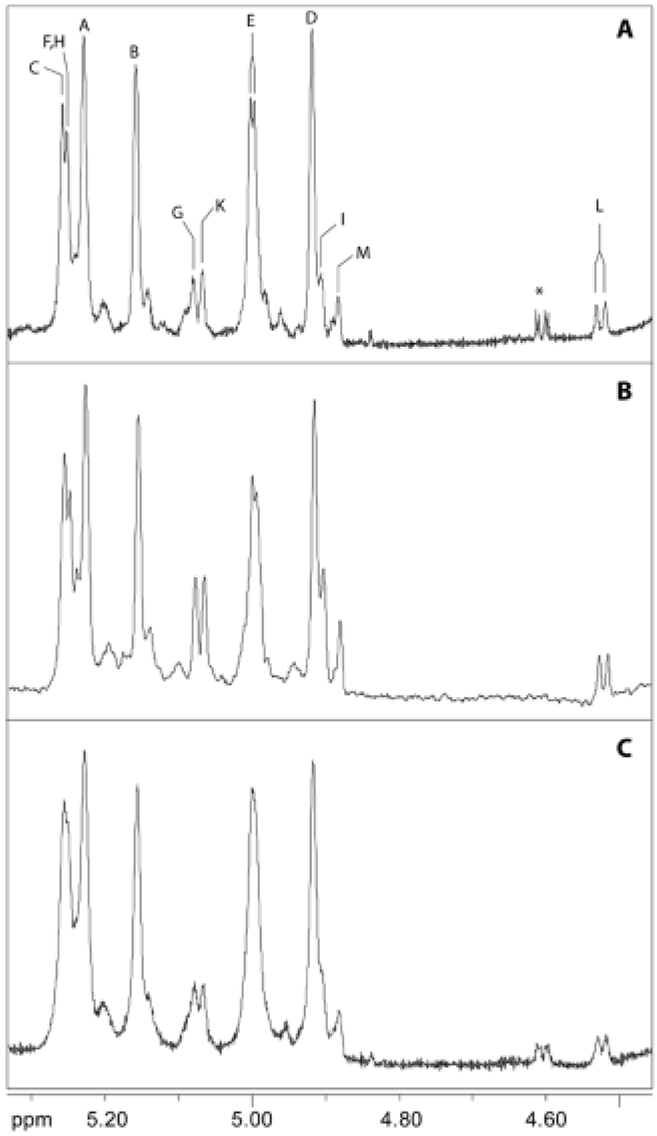

Supplemental Figure S2. Anomeric region of the 1-D Proton spectra of the three de-O-acetylated OPS samples. Panel A, STy-Gluc; **Panel B**, BRD948; **Panel C**, STy-FM. The signal marked with an asterisk could not be identified, but does not belong to an anomeric proton.

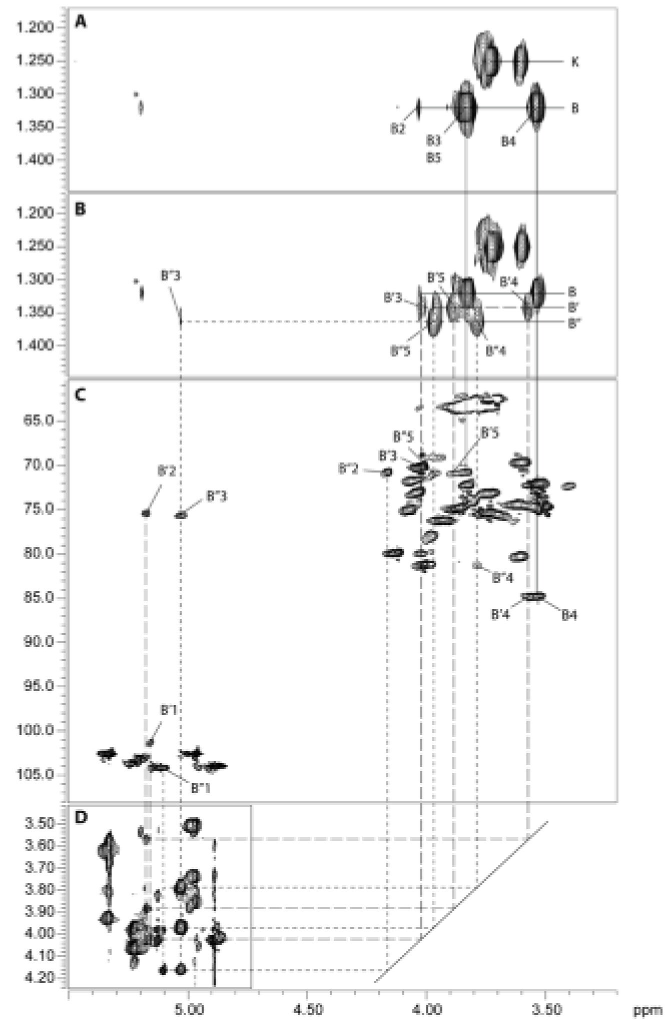

Supplemental Figure S3. NMR analysis of *S*. Typhi LPS. Panel A, Partial TOCSY spectrum of STy-Gluc, which lacks acetylation, showing cross peaks correlated to H-6 of 6-deoxysugars. Only one rhamnose (B) and one tyvelose (K) residue were found. The peak labeled with an asterisk is a zero-quantum NOESY correlation resulting from a strong NOE contact between H-6 of Residue B and H-1 of Residue A; **Panel B**, Partial TOCSY spectrum of BRD948, showing cross peaks correlated to H-6 of 6-deoxysugars. Two additional rhamnose residues (B' and B") were found. Solid lines connect peaks belonging to Residue B, long-dashed lines peaks of Residue B', and short-dashed lines peaks of Residue B"; **Panel C**, Partial HSQC spectrum of STy-FM. Only peaks of the acetylated rhamnose residues B' and B" are labeled; **Panel D**, Partial TOCSY spectrum of STy-FM, showing cross peaks of the anomeric signals. Together, the data demonstrate that both of the acetylated positions are part of two different rhamnose spin systems.

Supplemental References

1. Brett PJ, Burtnick MN, Snyder DS, Shannon JG, Azadi P, Gherardini FC. 2007. Burkholderia mallei expresses a unique lipopolysaccharide mixture that is a potent activator of human Toll-like receptor 4 complexes. Mol Microbiol 63:379-90.
2. York WS, Darvill AG, Mcneil M, Stevenson TT, Albersheim P. 1986. Isolation and Characterization of Plant-Cell Walls and Cell-Wall Components. Methods in Enzymology 118:3-40.
3. Anumula KR, Taylor PB. 1992. A comprehensive procedure for preparation of partially methylated alditol acetates from glycoprotein carbohydrates. Anal Biochem 203:101-8.
4. Wishart DS, Bigam CG, Yao J, Abildgaard F, Dyson HJ, Oldfield E, Markley JL, Sykes BD. 1995. 1H, 13C and 15N chemical shift referencing in biomolecular NMR. J Biomol NMR 6:135-40.
5. Hellerqvist CG, Lindberg B, Svensson S, Holme T, Lindberg AA. 1969. Structural studies on the O-specific side chains of the cell wall lipopolysaccharides from Salmonella typhi and S. enteritidis. Acta Chem Scand 23:1588-96.
6. Rahman MM, Guard-Petter J, Carlson RW. 1997. A virulent isolate of Salmonella enteritidis produces a Salmonella typhi-like lipopolysaccharide. J Bacteriol 179:2126-31.
7. Hone DM, Harris AM, Chatfield S, Dougan G, Levine MM. 1991. Construction of genetically defined double aro mutants of Salmonella typhi. Vaccine 9:810-6.
8. Tsai SP, Hartin RJ, Ryu J. 1989. Transformation in restriction-deficient Salmonella typhimurium LT2. J Gen Microbiol 135:2561-7.

9. Datsenko KA, Wanner BL. 2000. One-step inactivation of chromosomal genes in Escherichia coli K-12 using PCR products. Proc Natl Acad Sci U S A 97:6640-5.

10. Cherepanov PP, Wackernagel W. 1995. Gene disruption in Escherichia coli: TcR and KmR cassettes with the option of Flp-catalyzed excision of the antibiotic-resistance determinant. Gene 158:9-14.

11. Hasan N, Koob M, Szybalski W. 1994. Escherichia coli genome targeting, I. Cre-lox-mediated in vitro generation of ori- plasmids and their in vivo chromosomal integration and retrieval. Gene 150:51-6.

12. Chan W, Costantino N, Li R, Lee SC, Su Q, Melvin D, Court DL, Liu P. 2007. A recombineering based approach for high-throughput conditional knockout targeting vector construction. Nucleic Acids Res 35:e64.
